# SCD and MTHFD2 inhibitors for high‐risk acute myeloid leukaemia patients, as suggested by ELN2017‐pathway association

**DOI:** 10.1002/ctm2.1311

**Published:** 2023-06-28

**Authors:** Han Sun Kim, Doyeon Kim, Jiwoo Kim, Sunghyouk Park, Arvie Camille V. de Guzman

**Affiliations:** ^1^ Natural Products Research Institute College of Pharmacy, Seoul National University Seoul South Korea

Dear Editor,

The European LeukemiaNet (ELN) 2017 criteria^1^ is widely accepted as the risk classification of acute myeloid leukaemia (AML) patients. However, their application to studying risk‐related biological pathways is limited, failing to enhance treatment options for high‐risk patients. Using multiomics databases, biological pathways and genes whose upregulations correlate with increased ELN2017 risks and poorer survival were investigated, followed by experimental and functional validation, exhibiting SCD and MTHFD2 inhibitors as potential therapeutics for treating high‐risk AML patients.

As shown in Figure 1A, we initially evaluated ELN2017 and its revision^2^ for prognostic risk categorisation in OHSU patient data.^3^ Survival prognoses showed significant disparity among risk groups (Figures 1B and S1). Specifically, the ‘Very Adverse’ group exhibited a much poorer prognosis than other groups, consistent with a previous report.^2^ However, the ‘Very Favourable’ and ‘Favourable’ groups did not reveal significant differences (Table S1). This analysis shows four distinguished survival risk groups existing in OHSU data.

**FIGURE 1:**
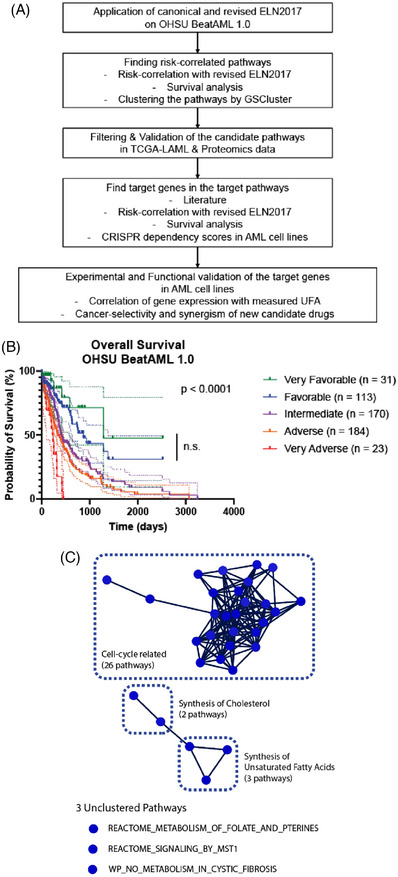
Overview of the study, application of revised ELN2017 to the OHSU database, and clustering for risk‐correlated biological pathways. (A) Overall scheme of the study. (B) Kaplan–Meier curves with 95% confidence intervals (dotted lines) for overall survival of AML patients in the OHSU BeatAML 1.0 database, according to the revised ELN2017 criteria. *p* value is from the log‐rank test. n.s. refers to not significant. Complete comparisons among the groups are in Table S1. (C) Clustering result of 34 pathways filtered by risk‐correlation with revised ELN2017 and survival analysis (see main text for details).

We then performed correlational screening between revised ELN2017 risks and pathway scores (FDR < 0.05), resulting in 690 pathways. Further refinement based on disease‐specific survival (DSS) criteria (FDR < 0.05, hazard ratio (HR) > 3, and FDR of HR < 0.05) resulted in 34 pathways. These pathways were categorised into three distinct clusters and three unclustered pathways (Figure 1C). The largest cluster (26 pathways) was ‘cell‐cycle related’; considering 71.2% (146/205) of the samples were from the initial diagnosis stage, a connection between the proliferative state at diagnosis and patient prognosis is suggested. As relapsed AML cells after chemotherapy exhibit higher dormancy^4^ and leukaemia stem cells often remain in a quiescent state,^5^ it will be an interesting future topic to specifically compare the relationship between the prognosis and the cell cycle progression at initial versus late‐stage AML. Since the ‘cell‐cyclerelated’ cluster already encompasses a standard regimen drug, cytarabine, we focused on other pathways (Figures 2A–D and S2–S4; Table 1).

**FIGURE 2:**
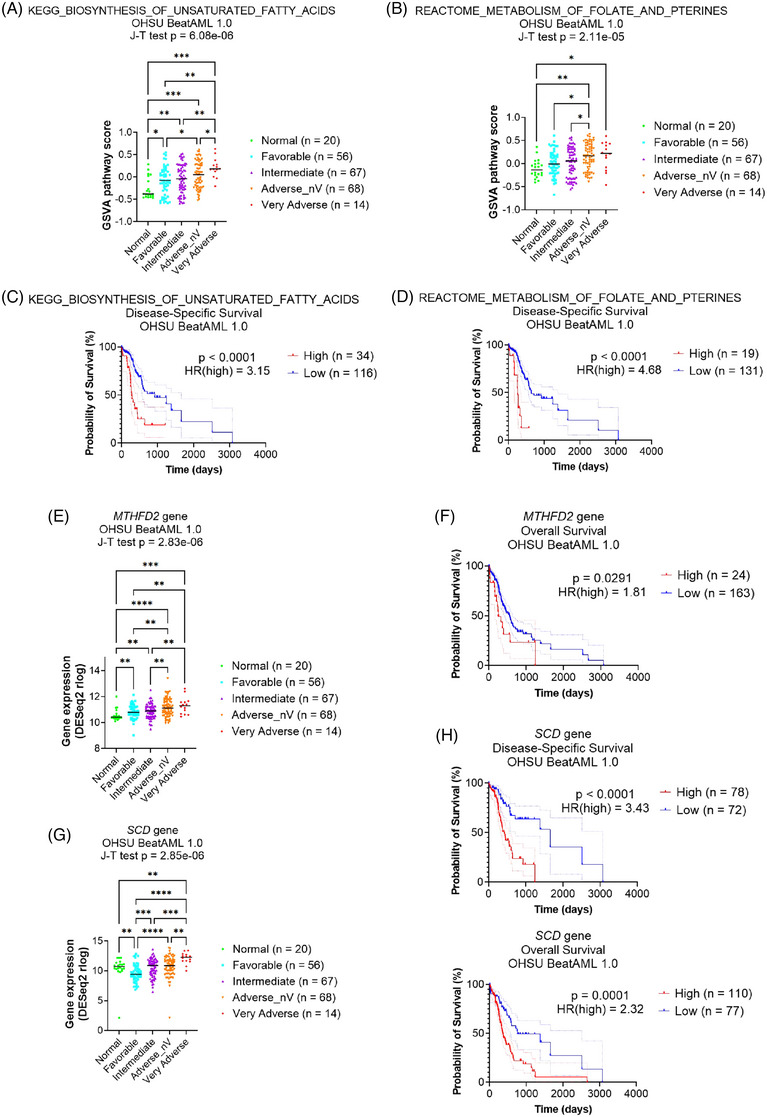
Identification of risk‐correlated biological pathways. The distributions of GSVA pathway scores of (A) KEGG_BIOSYNTHESIS_OF_UNSATURATED FATTY_ACIDS pathway or (B) REACTOME_METABOLISM_OF_FOLATE_AND_PTERINES pathway, in each risk category of revised ELN2017 in OHSU BeatAML 1.0 database. Kaplan–Meier curves with 95% confidence intervals (dotted lines) for disease‐specific survival of AML patients in OHSU BeatAML 1.0 database, for (C) KEGG_BIOSYNTHESIS_OF_UNSATURATED FATTY_ACIDS pathway or (D) REACTOME_METABOLISM_OF_FOLATE_AND_PTERINES pathway. The distributions of gene expression of (E) *MTHFD2* gene or (G) *SCD* gene in each risk category of revised ELN2017 in OHSU BeatAML 1.0 database. Kaplan–Meier curves with 95% confidence intervals (dotted lines) for (F) overall survival of AML patients in the OHSU BeatAML 1.0 database for *MTHFD2* gene or (H) disease‐specific survival and overall survival of AML patients in OHSU BeatAML 1.0 database for *SCD* gene. For (A), (B), (E) and (G), ‘Adverse_nV’ refers to the patients in the ‘Adverse’ category but not in the ‘Very Adverse’ category. The black lines indicate medians for each group. *p* values are from the Jonckheere‐Terpstra test. Post hoc analyses were performed with a two‐stage linear step‐up procedure. *p < .05, **p < .01, ***p < .001, ****p < .0001. For (C), (D), (F) and (H), *p* values are from the log‐rank test. The stratification of two groups in each graph was based on the best risk separation approach. HR(high) refers to the hazard ratio of the group with high pathway scores or gene expression.

**TABLE 1:** Risk‐correlation analysis of revised ELN2017 for pathways using multiomics databases.

	OHSU						
Pathway	J‐T test	Survival (DSS)	HR for DSS	Survival (OS)	HR for OS	TCGA‐LAML J‐T test	Proteomics J‐T test
KEGG_BIOSYNTHESIS_OF_UNSATURATED_FATTY_ACIDS (‘UFA_Synthesis’)	< 0.001	< 0.001	3.15	< 0.001	2.25	0.007	0.013
WP_OMEGA3OMEGA6_FA_SYNTHESIS	< 0.001	< 0.001	3.12	< 0.001	2.86	0.196	0.036
WP_OMEGA9_FA_SYNTHESIS	< 0.001	< 0.001	4.08	< 0.001	3.43	0.068	0.067
REACTOME_CHOLESTEROL_BIOSYNTHESIS	0.003	0.001	3.16	0.021	1.66	0.466	0.195
WP_MEVALONATE_PATHWAY	0.004	< 0.001	5.06	< 0.001	3.04	0.622	0.471
REACTOME_METABOLISM_OF_FOLATE_AND_PTERINES (‘Folate_Metabolism’)	< 0.001	< 0.001	4.68	< 0.001	2.35	0.006	0.001
REACTOME_SIGNALING_BY_MST1	< 0.001	< 0.001	3.22	< 0.001	2.81	< 0.001	NA
WP_NO_METABOLISM_IN_CYSTIC_FIBROSIS	0.002	< 0.001	3.28	0.006	2.30	< 0.001	0.220

J‐T test, Jonckheere‐Terpstra test; DSS, disease‐specific survival; OS, overall survival; HR, the hazard ratio of the high pathway score group; NA, not available.

Our correlations were further studied in two other large AML databases (TCGA‐LAML^6^ and proteomics database^7^) for eight pathways outside the ‘cell‐cyclerelated’ cluster. Patients were categorised into four groups using the revised ELN2017 as above. For the proteomics database, we leveraged Gene Set Variation Analysis (GSVA), originally used for transcriptomic data, to generate pathway scores. Combining analyses in all three databases revealed significant relationship between revised ELN2017 risk groups and ‘KEGG_BIOSYNTHESIS_OF_UNSATURATED_FATTY_ACIDS’ (‘UFA_Synthesis’) and ‘REACTOME_METABOLISM_OF_FOLATE_AND_PTERINES’ (‘Folate_Metabolism’) (Table 1).

After identifying risk‐correlated pathways, we examined targetable genes within those pathways. MTHFD2, a previously reported AML target,^8^, ^9^ was investigated for the Folate_Metabolism pathway. *MTHFD2* gene expression exhibited a significant increasing trend across ELN2017 risk groups (Figure 2E), and patients with higher gene expression experienced significantly shorter overall survival (Figure 2F). Clinical trials of methotrexate, an antifolate drug, were unsuccessful in AML due to reduced polyglutamylation activity, which is essential for its effectiveness.^10^ Therefore, alternative drugs targeting this pathway seem necessary.

For the UFA_Synthesis pathway, we analysed all 22 genes in the pathway in OHSU, TCGA‐LAML, and proteomics databases since little has been known for this pathway (Table S2). *ACOT7* (Figure S5) and *SCD* (Figure 2G and H) emerged as candidates. Among these, *SCD* exhibited the highest vulnerability upon knockout in AML cells (lowest median dependency score in the Depmap database; Figure S6). Therefore, we selected *SCD* as our target. Notably, while *SCD* level was higher in the ‘Normal’ group than in the ‘Favourable’ group, there was a clear and significant upward trend of *SCD* expression correlating with worse ELN2017 criteria (Figure 2G). This provides rationale for *SCD* as a target, aligning with our goal of finding targets for high‐risk AML patients.

With *MTHFD2* and *SCD* bioinformatically suggested as risk‐associated genes, we experimentally validated them using five AML (U937, MOLM‐14, THP‐1, KG‐1 and HL‐60) and one normal (HCC1954‐BL) cell line. CCK‐8 assay confirmed MTHFD2 inhibitor DS18561882 exhibited higher potency against all five AML cell lines compared to normal cell line (Figure 3A). SCD inhibitor A939572 also showed ∼8 times higher IC_50_s for normal cell line (Figure 3B). Trypan blue assay, along with normal peripheral blood mononuclear cells (PBMCs), also demonstrated the two inhibitors’ cancer cell selectivity (Figure S7).Additionally, SCD and MTHFD2 proteins’ expression was higher in AML cell lines than normal PBMCs (Figure S8). However, no significant correlations were observed among AML cell lines (Figure S9). DS18561882 treatment reduced MTHFD2 protein levels (Figure S10A), and A939572 treatment reduced unsaturated fatty acid levels without affecting SCD protein levels (Figure S10B and C), confirming the targeted effects of both drugs on the target proteins. Due to limited references regarding SCD in AML, we further validated its functional relevance. Unsaturated fatty acids (PUFA and UFA) were measured in AML cell lines using nuclear magnetic resonance (NMR). Among the 22 genes, *SCD* highly correlated with unsaturated fatty acids (Figures 3C and S11; Table S3). Our findings suggest the proposed inhibitors’ selectivity toward AML cells, indicating their potential use for high‐risk AML groups.

**FIGURE 3:**
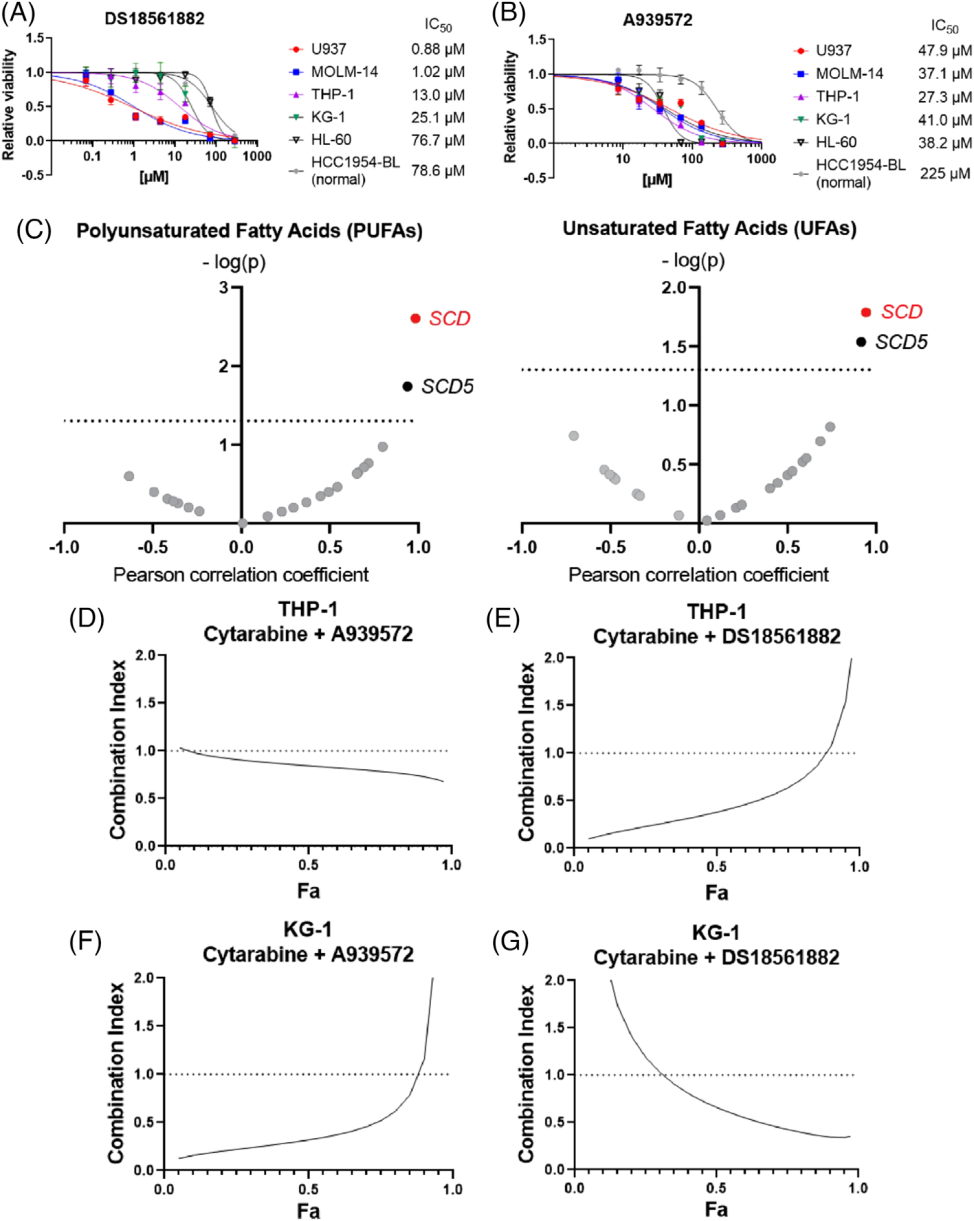
Experimental and functional validation of *SCD* and *MTHFD2* genes. Dose–response curves and IC_50_s for (A) DS18561882 drug and (B) A939572 drug to five AML cell lines (U937, MOLM‐14, THP‐1, KG‐1 and HL‐60) and one normal cell line (HCC1954‐BL) by CCK‐8 assay. The drugs were treated for 48 h. (C) Volcano plots for PUFAs and UFAs in the 5 AML cell lines identical to (A) and (B). The *x*‐axis refers to the Pearson correlation coefficient calculated by correlating gene expression of five AML cell lines in Depmap with measured PUFAs or UFAs by NMR for the genes only in the ‘UFA_Synthesis’ pathway. The *y*‐axis refers to the *p* value of the Pearson correlation coefficient. Nonsignificant (*p* value .05 or higher) genes are indicated in grey. Combination index plots for THP‐1 cell line for the combination of cytarabine with (D) A939572 or (E) DS18561882. Combination index plots for KG‐1 cell line for the combination of cytarabine with (F) A939572 or (G) DS18561882. For (D)–(G), the combination index of less than 1 indicates synergy and Fa refers to fractions affected by particular dose of a drug, herein the fraction of dead cells compared to nontreated samples.

The ‘cell‐cyclerelated’ cluster was found significantly correlated with AML‐risk groups. Cytarabine, a DNA replication inhibitor and standard‐of‐care drug for AML, is also used to high‐risk AML patients, along with hypomethylating agents and venetoclax regimen. When tested on cell lines, it exhibited a large variation in sensitivity (Figure S12). Combining A939572 or DS18561882 with cytarabine for cells with high cytarabine IC_50_ (THP‐1 and KG‐1; Figure S13) resulted in synergistic inhibition of cell survival (Figure 3D–G). In addition, dose reduction of cytarabine was observed in all four combinations (Table S4), suggesting the potential of A939572, DS18561882 or their derivatives in alleviating cytarabine toxicity.

Overall, our study identified risk‐associated pathways, target genes and potential synergistic drugs with cytarabine in AML through novel bioinformatic analysis on large multiomic datasets. Since not much is known about the roles of unsaturated fatty acids or folate metabolism in AML, our results could be further exploited to find a mechanistic relationship between those pathways and the malignancy of AML. More detailed discussion, including the significant advantage of our approach to finding new target genes/pathways (Table S5) or even in solid tumours, is in the Supplementary Information.

## AUTHOR CONTRIBUTIONS

HSK, DK and JK established the methodology, obtained the experimental data, performed the formal analysis and reviewed the manuscript. SP and ACVG conceptualised the study, obtained funding, supervised the study and wrote the manuscript.

## FUNDING

This work was supported by the Basic Science Research Program through the National Research Foundation of Korea grants funded by the Korean government Ministry of Science and ICT (NRF‐2018R1A3B1052328 to).

## CONFLICT OF INTEREST STATEMENT

The authors declare no potential conflicts of interest.

## Supporting information

Supporting Information

## Data Availability

For the original data, please contact arviecamille@snu.ac.kr.
