# Supplementary material for: SCD and MTHFD2 inhibitors for high‐risk acute myeloid leukaemia patients, as suggested by ELN2017‐pathway association
Source: Clin Transl Med. 2023 Jun 28;13(7):e1311. doi: 10.1002/ctm2.1311 (PMC10307990; doi:10.1002/ctm2.1311)
Supplement: Supplementary file 1 — Supporting Information [file CTM2-13-e1311-s001.docx]

**SCD and MTHFD2 inhibitors for high-risk acute myeloid leukemia patients, as suggested by ELN2017-pathway association**

Han Sun Kim, Doyeon Kim, Jiwoo Kim, Sunghyouk Park and Arvie Camille V. de Guzman

*Supplementary Data*

**Supplementary Methods**

- Databases used in the study

- Designation of canonical and revised ELN2017 risk criteria in the individual patients

- Transforming gene or protein expression data to pathway scores data

- Survival analysis and pathway clustering

- Cell lines and reagents

- UFA measurement by NMR

- Cell viability test and synergy test

- Western blotting

- Statistical analysis

**Supplementary Discussion**

**Supplementary Figure S1.**

**Supplementary Figure S2.**

**Supplementary Figure S3.**

**Supplementary Figure S4.**

**Supplementary Figure S5.**

**Supplementary Figure S6.**

**Supplementary Figure S7.**

**Supplementary Figure S8.**

**Supplementary Figure S9.**

**Supplementary Figure S10.**

**Supplementary Figure S11.**

**Supplementary Figure S12.**

**Supplementary Figure S13.**

**Supplementary Table S1.**

**Supplementary Table S2.**

**Supplementary Table S3.**

**Supplementary Table S4.**

**Supplementary Table S5.**

**References**

**Supplementary Methods**

**Databases used in the study**

For OHSU BeatAML 1.0 database^1^, gene expression data were obtained as follows; the raw counts were downloaded from the National Cancer Institute Genomic Data Commons (GDC, <https://portal.gdc.cancer.gov/>, downloaded on Nov. 2019) and summarized in a table, DESeq2-normalized and rlog-transformed using ‘DESeq2’ R package. Patients’ clinical information was obtained from the original paper^1^ and Vizome site (<http://vizome.org/>). For the information on the disease stage of the specimen, we referred to the “SpecimenGroups” parameter.

For TCGA-LAML database^2^, patients’ pancancer gene expression data and clinical information were downloaded from <https://gdc.cancer.gov/about-data/publications/pancanatlas> (downloaded on Feb. 2020). Gene expression data with only tumor samples retrieved and Entrez gene IDs were matched with gene symbols using the information downloaded from the National Center for Biotechnology Information (NCBI, <https://www.ncbi.nlm.nih.gov/>, downloaded on Sep. 2020). TCGA-LAML had no normal samples. The values in the expression table were added by 2, followed by log2-transformation. Then, only the gene expression with sample IDs starting with ‘TCGA-AB,’ which means they are the samples from the TCGA-LAML cohort, were retrieved. TP53 mutation status was retrieved from cBioPortal (<https://www.cbioportal.org/>) and karyotype information from GDC.

For AML Proteomics database^3^, tandem-mass-tag (TMT) protein abundance data and patients’ clinical information were downloaded from the site <https://proteomics.leylab.org/> (downloaded on Sep. 2022).

For the cell lines database, dependency scores in CRISPR screening and gene expression data were downloaded from Depmap (21Q2, <https://depmap.org/>). The designation of cell lines which are AML, was based on the metadata from the same database.

**Designation of canonical and revised ELN2017 risk criteria in the individual patients**

Different approaches were used depending on the databases for the designation of samples to canonical and revised ELN2017 risk groups. For OHSU BeatAML 1.0 database, two files contained clinical information; one is from the Vizome site (<http://vizome.org/>), and the other is from the original paper^1^. Since the two files were complementary, we used the two files as needed. Samples with the ‘ELN2017’ parameter as ‘Healthy, Individual BM MNC’ were designated as ‘Normal’ samples. Samples with ‘Favorable,’ ‘Intermediate,’ and ‘Adverse’ were designated as is. We only used samples from bone marrow aspirate, except for Fig. 1. Also, by manually reviewing the ‘specificDxAtAcquisition’ parameter, samples from non-AML patients were excluded. Among the ‘Favorable’ samples, those who have ‘inv(16)’ in the ‘specificDxAtAcquisition’ parameter or ‘y’ in the ‘CEBPA_Biallelic’ parameter were designated as ‘Very Favorable.’ Among the ‘Adverse’ samples, firstly, those with *TP53* mutation were found by excluding those who have ‘negative’ or NA as ‘TP53’ parameter; among them, by manually reviewing the parameter ‘Karyotype,’ samples only with complex karyotype were left to be designated as ‘Very Adverse.’ In total, the number of samples per each group was as follows; for survival analysis in Fig. 1, ‘Very Favorable’ (n = 31), ‘Favorable’ (n = 113), ‘Intermediate’ (n = 170), ‘Adverse’ (n = 184), and ‘Very Adverse’ (n = 23); for testing trends in Figs. 2 and 3, ‘Normal’ (n = 20), ‘Favorable’ (n = 56), ‘Intermediate’ (n = 68), ‘Adverse_nV’ (n = 68), and ‘Very Adverse’ (n = 14).

For the TCGA-LAML database, the designation of samples to canonical ELN2017 risk groups was kindly provided by the authors of the previous publication^4^. For the designation of the ‘Very Adverse’ category, we utilized *TP53* mutation status information from cBioPortal, and karyotype information from GDC. The samples having ‘Complex’ in the ‘cytogenetic_abnormality_type’ parameter were regarded as having complex karyotypes, and the samples which both have *TP53* mutation and complex karyotype were designated as ‘Very Adverse.’ In total, the number of samples per each group was as follows; ‘Favorable’ (n = 59), ‘Intermediate’ (n = 38), ‘Adverse_nV’ (n = 45), and ‘Very Adverse’ (n = 11).

For the AML Proteomics database, canonical ELN2017 risk groups were designated according to the ‘RISK (ELN2017)’ parameter. The samples which do not have NA as the ‘TP53’ parameter were regarded as having *TP53* mutation, and among them, we determined complex karyotype by reviewing the ‘Cytogenetics’ parameter; those having both *TP53* mutation and complex karyotype were designated as ‘Very Adverse.’ In total, the number of samples per each group was as follows; except for the *SCD* gene, ‘Favorable’ (n = 14), ‘Intermediate’ (n = 10), ‘Adverse_nV’ (n = 16), and ‘Very Adverse’ (n = 4); for *SCD* gene, ‘Favorable’ (n = 3), ‘Intermediate (n = 3)’, ‘Adverse_nV’ (n = 10), and ‘Very Adverse’ (n = 2).

In all databases, ‘Adverse_nV’ samples were designated by excluding ‘Very Adverse’ samples from ‘Adverse’ samples.

**Transforming gene or protein expression data to pathway scores data**

‘GSVA’ R package^5^ was used to convert gene or protein expression data to pathway scores data. For input for pathways, the combination of curated canonical pathways (which includes pathways from BioCarta, KEGG, PID, Reactome, and WikiPathways) and hallmark gene sets from MSigDB (version 7.2) was used. The R package ‘GSA’ was used when reading the gene sets to R. For parameters when running GSVA, ‘min.sz’ was set to 5, ‘max.sz’ to 700, and ‘method’ to ‘gsva.’

**Survival analysis and pathway clustering**

For survival analysis, ‘survival’ and ‘survminer’ R packages were used. When conducting pairwise comparisons among more than two survival curves, the pairwise_survdiff function was used with p.adjust.method parameter set to ‘fdr.’ The stratification of two groups by gene expression or pathway scores was based on a minimum p-value approach^6^, using the surv_cutpoint function with the minprop parameter set to 0.1. Log-rank test and Cox regression were used to compare the curves and obtain hazard ratios using GraphPad Prism 9.1.1 (GraphPad Software, San Diego, CA, USA) or R (version 4.1.1).

For disease-specific survival (DSS) analysis in the OHSU database, only patients with the ‘causeOfDeath’ parameter as ‘Dead-Disease’ and ‘Alive’ were included. For overall survival (OS) analysis in the OHSU database, patients who did not have the parameter as NA were included.

For the clustering of pathways, ‘GSCluster’ R package^7^ was used. For the q value input for GSCluster, FDR values from the Jonckheere-Terpstra test screening result were used. When plotting the clusters, the Maximum gene-set distance parameter was set to 0.85.

**Cell lines and reagents**

MOLM-14 (DSMZ, Braunschweig, Germany), U937, THP-1, KG-1, HCC1954-BL (ATCC, Manassas, VA, USA), and HL-60 (Korean Cell Line Bank, Seoul, Korea) were used in this study. Cryopreserved peripheral blood mononuclear cells (PBMCs) were purchased from Zen-Bio (Research Triangle, NC, USA). Cells were cultured or incubated in RPMI 1640 media supplemented with 10% fetal bovine serum (FBS) (for KG-1 only, 20%), 100 U/mL penicillin, and 100 μg/mL streptomycin, at 37 °C in a 5% CO2 incubator. A939572 and DS18561882 were purchased from MedChemExpress (Monmouth Junction, NJ, USA). Cytarabine was purchased from Sigma-Aldrich (St.Louis, MO, USA).

**UFA measurement by NMR**

U937, MOLM-14, THP-1, KG-1, and HL-60 cell lines were seeded 1 × 10^7^ cells in 10 mL of media and cultured for 24 hours; to minimize potential factors affecting UFA amount in the cells, the media was unified to RPMI 1640 media supplemented with 20% fetal bovine serum (FBS), 100 U/mL penicillin, and 100 μg/mL streptomycin. For Supplementary Fig. S10C, U937 cells were seeded 2 × 10^6^ cells in 6 mL of media and cultured for 48 hours. Here, RPMI 1640 media supplemented with 10% fetal bovine serum (FBS), 100 U/mL penicillin, and 100 μg/mL streptomycin was used to make the media consistent with Supplementary Fig. S10B. The cells were harvested and underwent standard two-phase extraction, and the lipid phase was dried by speedvac. The dried samples were dissolved in chloroform-d_6_ (Cat. 151823, Sigma-Aldrich) and subjected to 1H NMR. 800 MHz Bruker Avance III HD spectrometer equipped with a 5 mm CPTCI CryoProbe (Bruker BioSpin, Germany) was used. The spectra were processed with MestReNova software (version 12.0.1-20560). The UFA and PUFA amounts were measured as the area under 5.29-5.40 ppm and 2.70-2.90 ppm of the spectrum, respectively. Then, for each cell line, the numbers were normalized by the cell numbers counted when the cells were harvested, respectively.

**Cell viability test and synergy test**

For CCK-8 assay, cells were seeded at 1 × 10^4^ cells/well on 96-well plates and treated with drugs (A939572, DS18561882, Cytarabine) at various concentrations for 48 hours, and measured with the D-plus CCK Cell Viability Assay Kit (Dongin Biotech, Seoul, South Korea). For Trypan blue assay, 1-2 × 10^4^ cells/well (for cell lines) or 1 × 10^5^ cells/well (for PBMCs) on 96-well plates and treated with drugs (A939572, DS18561882) at various concentrations for 48 hours. The cells were counted with Countess II FL Automated Cell Counter (Thermo Fisher Scientific, Waltham, USA) or hemocytometer (for PBMCs). The IC_50_ value was obtained using the GraphPad Prism 9.1.1 software. The test for synergy and calculation of dose reduction indices were done using CompuSyn software^8^.

**Western blotting**

The samples were homogenized in T-PER^TM^ Tissue Protein Extraction Reagent (Thermo Scientific, USA) buffer with protease and phosphatase inhibitors (1 mM PMSF (phenylmethylsulfonyl fluoride) 2 µg/mL aprotinin, 1 µg/mL pepstatin A). 20 or 30 µg of protein extracts were loaded, and they were separated by SDS electrophoresis with 10% gel, then transferred to the NC (nitrocellulose) membranes. Membranes were blocked with 5% skim milk in TBST (Tris-buffered saline with 0.1% tween) and incubated at 4℃ overnight with the following primary antibodies: β-actin (sc-47778, Santa Cruz), SCD (A16429, Abclonal), MTHFD2 (A22653, Abclonal). Anti-rabbit IgG-HRP (31460, Invitrogen) and anti-mouse IgG-HRP (31430, Invitrogen) were used as secondary antibodies. The protein bands were visualized by using a Westsave star kit (Abfrontier, Seoul, South Korea) and imaged on a Fusion Solo Chemi-DOC (Vilber Lourmat, France). The quantification of the bands was done with EvolutionCapt software (Vilber Lourmat, France).

**Statistical analysis**

Jonckheere-Terpstra test was used when testing for increasing trends across groups, using the ‘DescTools’ R package. Since one of the assumptions for the Jonckheere-Terpstra test is that the observations should be independent, ‘Adverse_nV’ instead of ‘Adverse’ was used when performing the test. Post hoc analyses were done with the two-stage linear step-up procedure of Benjamini, Krieger, and Yekutieli in GraphPad Prism. For Supplementary Fig. S10C, Student’s *t*-test was used. Details are in the ‘Survival analysis and pathway clustering’ section for survival analysis. For correlation analysis, Pearson’s correlation was used. All statistical analyses were performed with GraphPad Prism 9.1.1 or R (version 4.1.1).

**Supplementary Discussion**

Since its inception in 2017, ELN2017 has been used for diagnosing and managing AML patients and has also been applied to research. Still, most studies have focused on validating ELN2017 risk criteria in terms of survival in individual hospitals, and only a few papers have addressed specific biological pathways dysregulated in the high-risk groups or specific drugs targeting these pathways. Some focused on individual mutations comprising the ‘Adverse’ category of ELN2017, such as *RUNX1* mutation^9^ or *TP53* mutation^10^. Another study found gene modules related to 14 markers, including ELN2017 and the contributing mutations, later focusing on modules correlated with *NPM1*/*FLT3*-ITD mutation^11^. Other researchers first sought to find particular genes related to survival and then constructed the prognostic risk scores correlated with ELN2017^12,13^. Of note, one of the studies found that the high-risk phenotype score is enriched with the biosynthesis of unsaturated fatty acid gene set^12^, consistent with our result implicating high expression of the *SCD* gene in high-risk groups. Furthermore, a metabolomics study also showed that plasma levels of some species of PUFA were positively correlated with risk stratification^14^ in AML patients.

Compared with the previously reported approaches, the critical difference in our current work is that we initiated the analysis by assessing pathways that directly correlate with ELN2017 using GSVA pathway scores. Other studies started with individual genes, rather than pathways, for survival relationships or focused on individual mutations comprising ELN2017. Then, we subsequently validated the resulting pathways and genes with survival data and also with results from other multiomics databases. We also added the ‘Very adverse’ group and the ‘normal’ group, which were not included in most of the prior studies, which, we believe, gave more reliable results. We also utilized the proteomics AML database, which has not been used in the above studies, adding confidence to our results. Probably most importantly, we carried out experimental and functional validation of the target genes after the bioinformatics screening, which is seldom the case for most related studies. Our results showing the relationship between *SCD* expression and actual UFA and PUFA amounts suggest that *SCD* is a promising target for AML. We believe that these extra steps enabled our suggested drugs, A939572 and DS18561882, to exhibit selectivity toward cancer cells and efficacy against refractory AML cells. These drugs’ synergy and dose reduction for cytarabine against cell lines with high IC_50_ values are also noteworthy for developing new combinational treatments. To our knowledge, only limited information about SCD inhibitors on AML is available. For MTHFD2 inhibitors, their effects on AML have been revealed only recently, particularly the study suggesting pyrimidine depletion and replication stress as the underlying mechanism^15^. Although the authors observed synergy between MTHFD2 and ATR or dUTPase inhibitors, they did not address the synergy with cytarabine, a standard-of-care drug. Our results for synergy between SCD/MTHFD2 inhibitors and cytarabine may have additional implications in this respect.

Furthermore, the significant advantage of our approach is that it can also be applied to find other target genes/pathways, or even for solid tumors. Even though we chose the *MTHFD2* gene in the Folate_Metabolism pathway due to its literature evidence, when we looked for another candidate gene using the same approach used in the UFA_Synthesis pathway, *ALDH1L2* gene was found to be the only gene significant in all three databases (Supplementary Table S5). Unlike *MTHFD2*, in AML, no literature study and no inhibitors are available, but as seen in the case of the *SCD* gene, the *ALDH1L2* gene could be the possible target. In addition, in solid tumors, the ELN2017 category is analogous to stage or grade information in that it is closely related to patient prognosis. Other relevant parameters, such as recurrence status after initial treatment and lymph/distant metastasis status available in TCGA databases, could also be combined to build an ELN2017-like variable for prognosis categorization. Then, a simple Jonckheere-Terpstra test can be applied to correlate pathways to the variable, as it is a non-parametric test that can be used regardless of sample distributions. This correlation between the prognosis variable and pathways should give more valuable information related to cancer malignancy than conventional analysis of tumor vs. normal samples.

Very recently, a new version of ELN recommendation, ELN2022, was introduced^16^. While the stratification of patients into three categories was maintained, it now uses more information on patient genetics, such as bZIP in-frame mutated CEBPA, KAT6A::CREBBP fusion, or variant allele fraction of *TP53* mutation^16^, some of which information is not registered in the present databases. According to a study that attempted the validation of ELN2022 in clinic^17^, 83%, 72%, and 90% of patients in each risk category kept their allocation in ELN2017, suggesting largely similar categorization. In addition, ELN2022 did not perform better than ELN2017 in outcome prognostication. Therefore, our analysis with ELN2017 should still be meaningful and valuable even with the new version of risk classification.

**
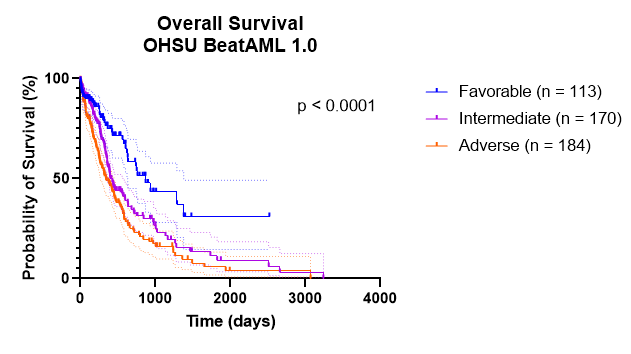
**

**Supplementary Figure S1. Application of canonical ELN2017 to the OHSU database**

Kaplan-Meier curves with 95% confidence intervals (dotted lines) for overall survival of AML patients in the OHSU BeatAML 1.0 database, according to the canonical ELN2017 criteria. *P*-value is from the log-rank test.

**
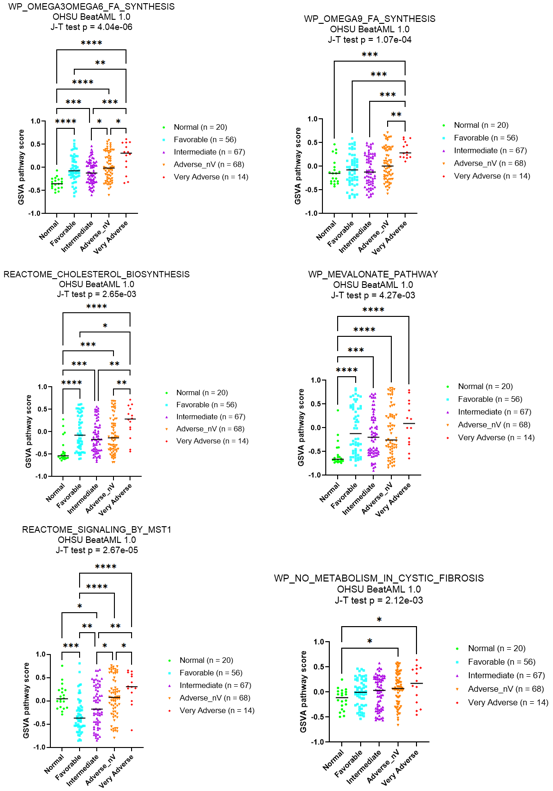
**

**Supplementary Figure S2. The distributions of GSVA pathway scores of high-risk pathways in the OHSU database.**

The GSVA pathway scores for each risk category of the revised ELN2017 are plotted. The pathways are from Fig. 1C, except for the pathways in the ‘cell-cycle related’ cluster, KEGG_BIOSYNTHESIS_OF_UNSATURATED FATTY ACIDS pathway and REACTOME_METABOLISM_OF_FOLATE_AND_PTERINES pathway. ‘Adverse_nV’ refers to patients in the ‘Adverse’ category but not the ‘Very Adverse’ category. The black lines indicate medians for each group. *P*-values are from Jonckheere-Terpstra test. Post hoc analyses were performed with a two-stage linear step-up procedure. * *p* < 0.05, ** *p* < 0.01, *** *p* < 0.001, **** *p* < 0.0001.

**
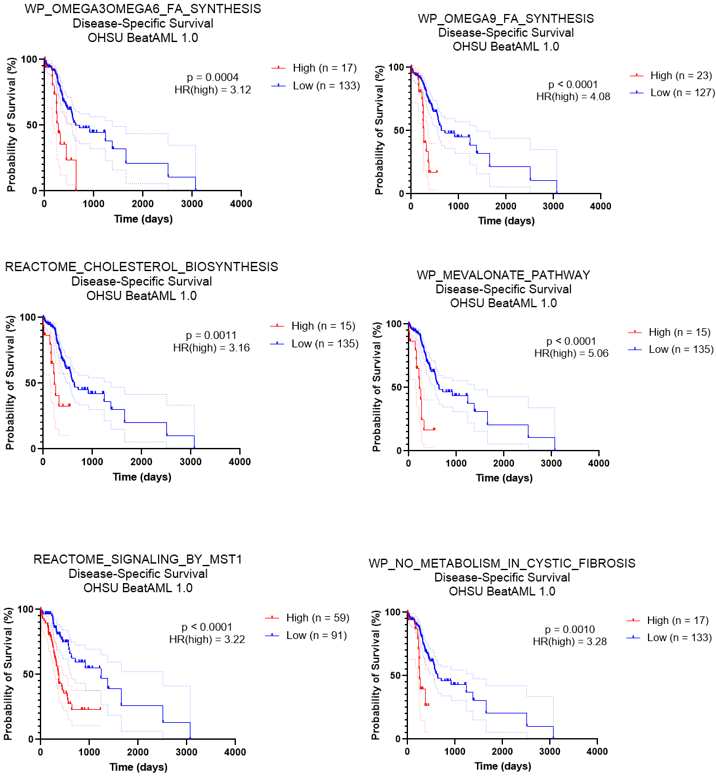
Supplementary Figure S3. Disease-specific survival analysis of high-risk pathways in the OHSU database.**

Kaplan-Meier curves with 95% confidence intervals (dotted lines) for disease-specific survival of AML patients in OHSU BeatAML 1.0 database for pathways in Fig. 1C, except for pathways in the ‘cell-cycle related’ cluster, KEGG_BIOSYNTHESIS_OF_UNSATURATED FATTY ACIDS pathway and REACTOME_METABOLISM_OF_FOLATE_AND_PTERINES pathway. *P*-values are from the log-rank test. The stratification of two groups in each graph was based on the best risk separation approach. HR(high) refers to the hazard ratio of the group with high pathway scores.

**
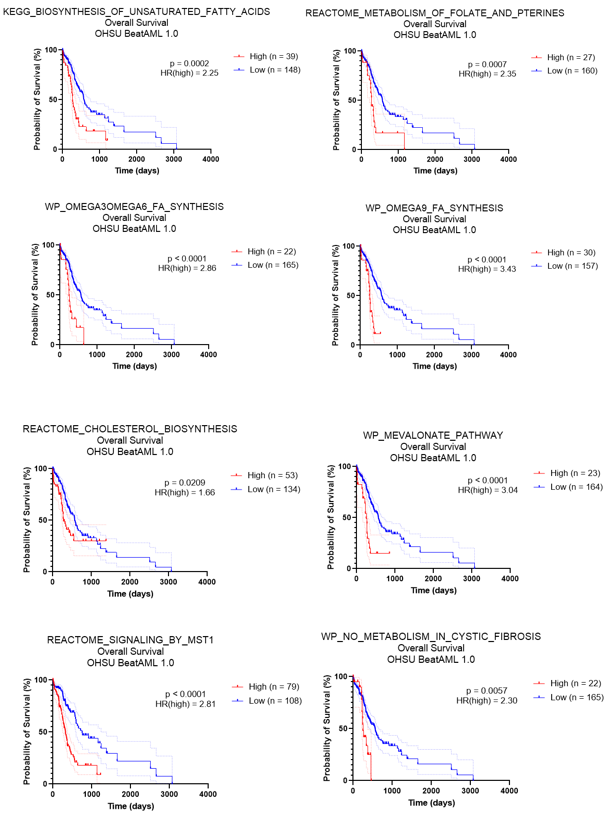
Supplementary Figure S4. Overall survival analysis of high-risk pathways in OHSU database.**

Kaplan-Meier curves with 95% confidence intervals (dotted lines) for overall survival of AML patients in the OHSU BeatAML 1.0 database for pathways in Fig. 1C, except for pathways in the ‘cell-cycle related’ cluster. *P*-values are from the log-rank test. The stratification of two groups in each graph was based on the best risk separation approach. HR(high) refers to the hazard ratio of the group with high pathway scores.

**
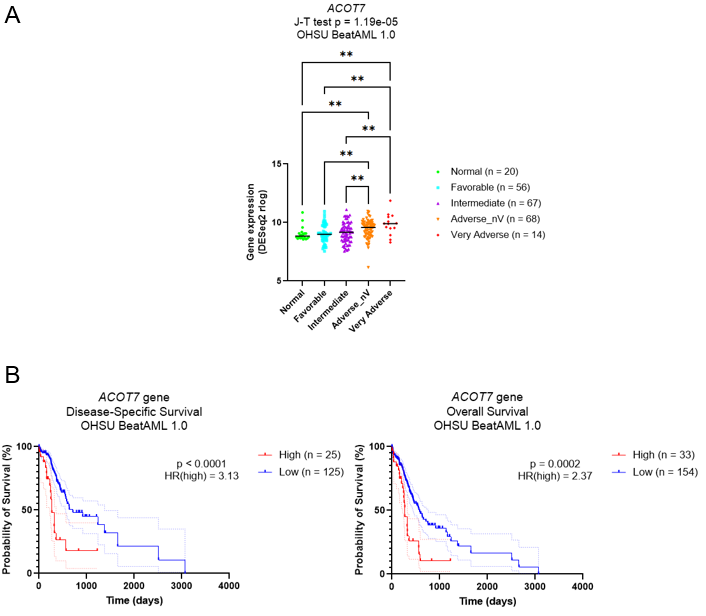
**

**Supplementary Figure S5. Risk-correlation and survival analysis for ACOT7 gene in the OHSU database.**

(A) The distributions of gene expression of the *ACOT7* gene in each risk category of revised ELN2017 in the OHSU BeatAML 1.0 database. ‘Adverse_nV’ refers to patients in the ‘Adverse’ category but not the ‘Very Adverse’ category. The black lines indicate medians for each group. *P*-value is from the Jonckheere-Terpstra test. Post hoc analyses were performed with a two-stage linear step-up procedure. * *p* < 0.05, ** *p* < 0.01. (B) Kaplan-Meier curves with 95% confidence intervals (dotted lines) for disease-specific survival and overall survival of AML patients in the OHSU BeatAML 1.0 database for the *ACOT7* gene. *P*-values are from the log-rank test. The stratification of two groups in each graph was based on the best risk separation approach. HR(high) refers to the hazard ratio of the group with high expression.

**
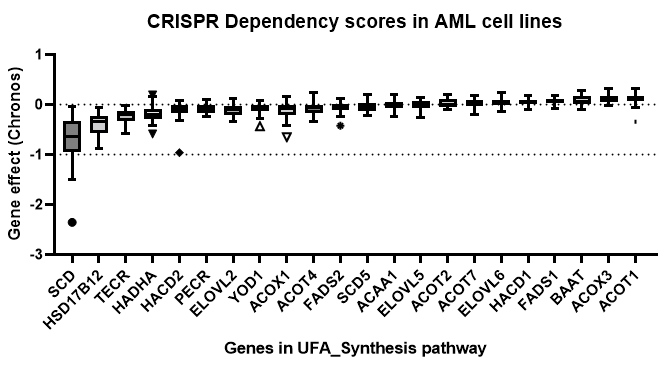
**

**Supplementary Figure S6. Identifying essentiality for genes in the ‘UFA_Synthesis’ pathway in AML.**

The distributions of CRISPR dependency scores in AML cell lines for each gene comprising the ‘UFA_Synthesis’ pathway. The scores were retrieved from Depmap. A score of 0 represents no viability effect, and a score of -1 corresponds to the median effect of known common-essential genes. A lower score indicates a higher likelihood that the gene of interest is essential in the given cell line. The genes are ordered by medians of the scores.

**
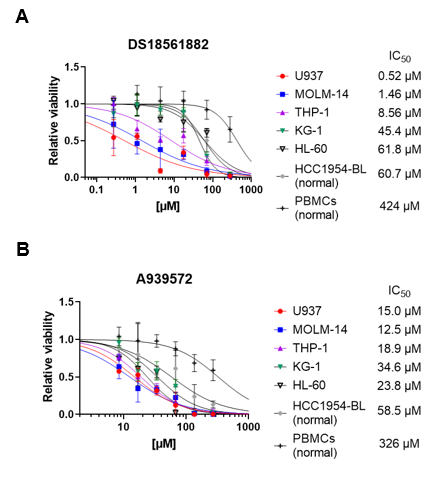
**

**Supplementary Figure S7. Cell viability assays, including PBMCs.**

Dose-response curves and IC_50_s for (A) DS18561882 and (B) A939572 to five AML cell lines (U937, MOLM-14, THP-1, KG-1, and HL-60), one normal cell line (HCC1954-BL) and normal PBMCs by Trypan blue assay. The drugs were treated for 48 hours.

**
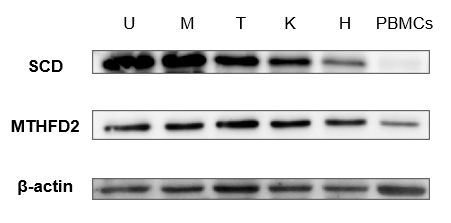
**

**Supplementary Figure S8. Basal SCD and MTHFD2 protein expression in AML cell lines and PBMCs.**

Western blot for SCD and MTHFD2 proteins in five AML cell lines (U937, MOLM-14, THP-1, KG-1, and HL-60) and normal PBMCs. U, M, T, K, and H refer to U937, MOLM-14, THP-1, KG-1, and HL-60, respectively.

**
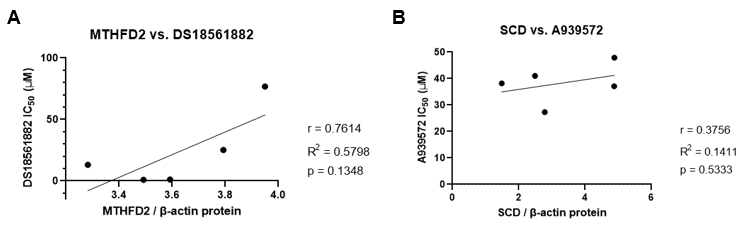
**

**Supplementary Figure S9. Correlation of MTHFD2 and SCD protein levels with their respective inhibitors.**

The relationship of (A) MTHFD2 protein level with DS18561882 IC_50_s and (B) SCD protein level with A939572 IC_50_s in the 5 AML cell lines (U937, MOLM-14, THP-1, KG-1 and HL-60). The IC_50_s are from Fig. 3A and B. Regression lines, r, R^2,^ and *p*-values from Pearson correlation analysis are shown.

**
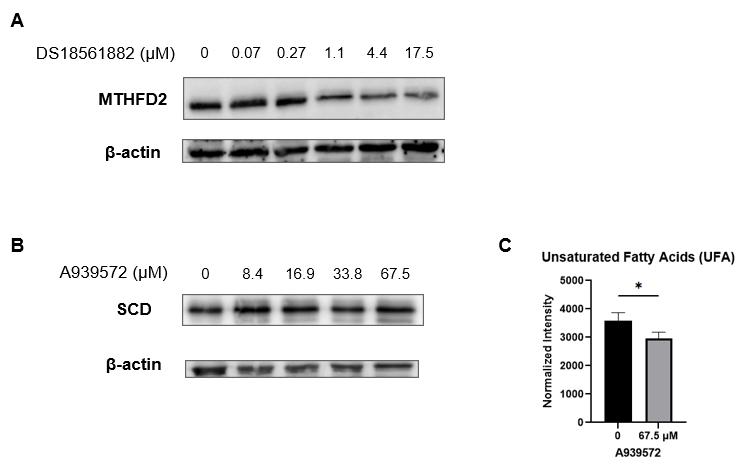
**

**Supplementary Figure S10. Effect of DS18561882 on MTHFD2 protein and A939572 on SCD protein.**

Western blot for (A) MTHFD2 protein in the MOLM-14 cell line treated with DS18561882 and (B) SCD protein in the U937 cell line in the indicated concentrations. The drugs were treated for 48 hours. (C) The amount of unsaturated fatty acids normalized by the cell numbers between A939572 non-treated and treated U937 cell lines. The drug was treated for 48 hours. *P*-value is from the Student’s *t*-test. * *p* < 0.05.

**
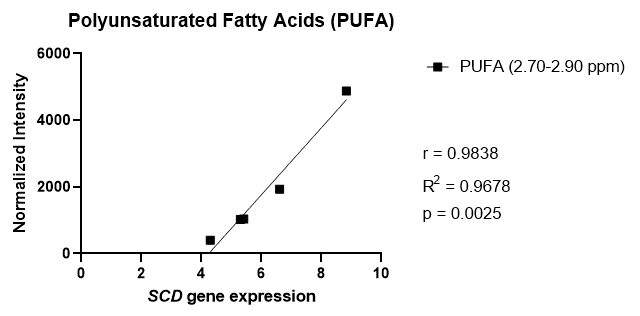
**

**
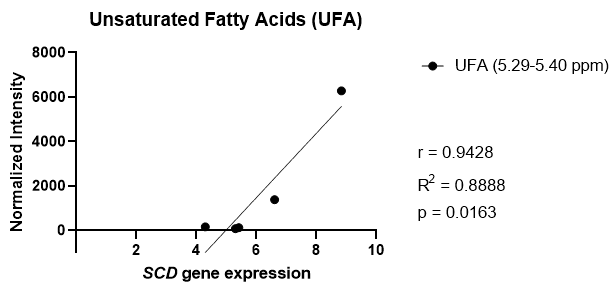
**

**Supplementary Figure S11. Functional validation of *SCD* gene.**

The relationship of *SCD* gene expression and measured PUFAs or UFAs in the 5 AML cell lines (U937, MOLM-14, THP-1, KG-1, and HL-60). Regression lines, r, R^2,^ and *p*-values from Pearson correlation analysis are shown.

**
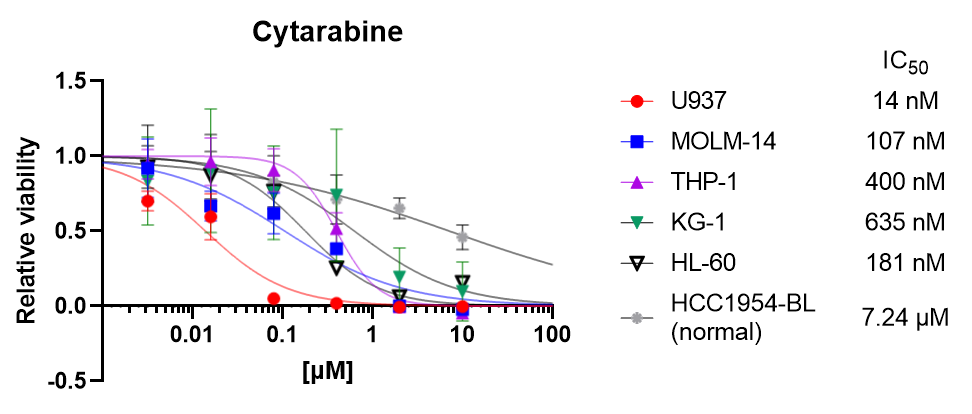
**

**Supplementary Figure S12. Dose-response curves for cytarabine.**

Dose-response curves and IC_50_s for cytarabine to five AML cell lines (U937, MOLM-14, THP-1, KG-1, and HL-60) and one normal cell line (HCC1954-BL). The drugs were treated for 48 hours.

**
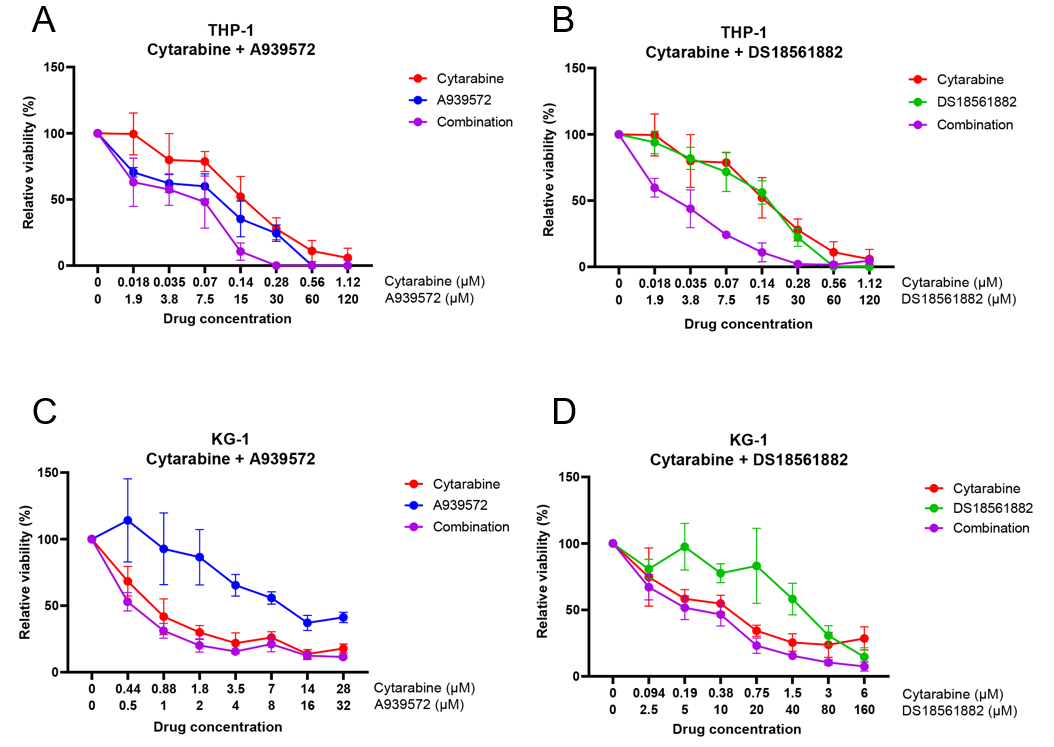
**

**Supplementary Figure S13. Synergy of SCD and MTHFD2 inhibitor with cytarabine.**

(A, B) Dose-response curves for THP-1 cell line, testing synergy of cytarabine either with (A) A939572 or (B) DS18561882. (C, D) Dose-response curves for KG-1 cell line, the testing synergy of cytarabine either with (C) A939572 or (D) DS18561882.

**Supplementary Table S1.**

Results of pairwise comparisons among all groups in Fig. 1B.

|  | Adverse | Favorable | Intermediate | Very Adverse |
| --- | --- | --- | --- | --- |
| Favorable | < 0.001 | - | - | - |
| Intermediate | 0.030 | 0.002 | - | - |
| Very Adverse | 0.002 | < 0.001 | < 0.001 | - |
| Very Favorable | < 0.001 | 0.152 | 0.003 | < 0.001 |

**Supplementary Table S2.**

Risk-correlation analysis of revised ELN2017 for genes in the UFA_Synthesis pathway using multiomics databases.

| **Gene** |  | **OHSU** | | | |  | **TCGA-LAML** | **Proteomics** |
| --- | --- | --- | --- | --- | --- | --- | --- | --- |
|  | Jonckheere-Terpstra test | | Survival (DSS) | HR | Survival (OS) | HR | Jonckheere-Terpstra test | Jonckheere-Terpstra test |
| *ACAA1* | 0.940 | | 0.076 | 0.582 | 0.119 | 0.683 | 1.000 | 0.929 |
| *ACOT1* | < 0.001 | | 0.199 | 0.702 | 0.267 | 0.785 | 0.102 | 0.325 |
| *ACOT2* | < 0.001 | | 0.210 | 1.410 | 0.197 | 0.624 | 0.005 | 0.834 |
| *ACOT4* | 0.420 | | 0.089 | 0.641 | 0.033 | 0.627 | 0.999 | 0.500 |
| *ACOT7* | < 0.001 | | < 0.001 | 3.130 | < 0.001 | 2.370 | 0.004 | 0.007 |
| *ACOX1* | 0.317 | | 0.023 | 0.551 | 0.079 | 0.693 | 0.652 | 0.364 |
| *ACOX3* | 0.329 | | 0.086 | 0.580 | 0.032 | 0.600 | 0.006 | 0.067 |
| *BAAT* | 0.014 | | < 0.001 | 3.090 | < 0.001 | 2.510 | 0.001 | NA |
| *ELOVL2* | 0.067 | | 0.004 | 2.090 | 0.005 | 1.780 | < 0.001 | NA |
| *ELOVL5* | 0.878 | | 0.005 | 0.413 | 0.016 | 0.530 | 0.023 | 0.013 |
| *ELOVL6* | 0.301 | | 0.004 | 2.710 | 0.106 | 1.800 | 0.001 | NA |
| *FADS1* | < 0.001 | | 0.002 | 2.640 | 0.004 | 1.990 | 0.020 | 0.114 |
| *FADS2* | 0.007 | | 0.048 | 1.800 | 0.048 | 1.610 | 0.084 | 0.013 |
| *HACD1* | 0.053 | | 0.005 | 2.070 | 0.029 | 1.610 | 0.576 | 0.977 |
| *HACD2* | 0.724 | | 0.019 | 1.870 | 0.009 | 1.720 | 0.001 | 0.021 |
| *HADHA* | 0.254 | | 0.056 | 0.617 | 0.027 | 0.628 | 0.966 | 0.542 |
| *HSD17B12* | 0.089 | | 0.049 | 1.770 | 0.068 | 1.500 | 0.895 | 0.774 |
| *PECR* | 0.389 | | < 0.001 | 2.930 | < 0.001 | 2.110 | < 0.001 | 0.001 |
| *SCD* | < 0.001 | | < 0.001 | 3.430 | < 0.001 | 2.320 | < 0.001 | 0.010 |
| *SCD5* | 0.656 | | 0.006 | 2.030 | 0.012 | 1.810 | 0.796 | 0.563 |
| *TECR* | 0.703 | | 0.083 | 1.870 | 0.073 | 1.480 | 0.238 | 0.252 |
| *YOD1* | 0.019 | | 0.029 | 2.000 | 0.046 | 1.650 | < 0.001 | 0.075 |

DSS, disease-specific survival; OS, overall survival; HR, the hazard ratio of the high expression group; NA, not available.

**Supplementary Table S3.**

UFA and PUFA correlation analysis for genes in the UFA_Synthesis pathway.

| **Gene** | **UFA (5.29 – 5.40 ppm)** | | **PUFA (2.70 – 2.90 ppm)** | |
| --- | --- | --- | --- | --- |
|  | Pearson’s r | *p*-value | Pearson’s r | *p*-value |
| *ACAA1* | 0.585 | 0.300 | 0.662 | 0.224 |
| *ACOT1* | -0.536 | 0.352 | -0.417 | 0.485 |
| *ACOT2* | -0.487 | 0.406 | -0.358 | 0.554 |
| *ACOT4* | -0.471 | 0.423 | -0.383 | 0.525 |
| *ACOT7* | 0.500 | 0.391 | 0.493 | 0.398 |
| *ACOX1* | -0.116 | 0.853 | 0.008 | 0.989 |
| *ACOX3* | 0.401 | 0.504 | 0.447 | 0.451 |
| *BAAT* | -0.505 | 0.386 | -0.493 | 0.399 |
| *ELOVL2* | -0.351 | 0.562 | -0.297 | 0.627 |
| *ELOVL5* | 0.741 | 0.152 | 0.798 | 0.106 |
| *ELOVL6* | -0.334 | 0.583 | -0.236 | 0.702 |
| *FADS1* | 0.527 | 0.362 | 0.653 | 0.232 |
| *FADS2* | 0.605 | 0.280 | 0.719 | 0.171 |
| *HACD1* | 0.209 | 0.736 | 0.367 | 0.543 |
| *HACD2* | 0.242 | 0.695 | 0.295 | 0.629 |
| *HADHA* | 0.685 | 0.202 | 0.693 | 0.195 |
| *HSD17B12* | -0.708 | 0.181 | -0.633 | 0.252 |
| *PECR* | 0.119 | 0.849 | 0.230 | 0.710 |
| *SCD* | 0.943 | 0.016 | 0.984 | 0.002 |
| *SCD5* | 0.916 | 0.029 | 0.938 | 0.018 |
| *TECR* | 0.044 | 0.944 | 0.149 | 0.811 |
| *YOD1* | 0.444 | 0.454 | 0.543 | 0.344 |

UFA, unsaturated fatty acid; PUFA, polyunsaturated fatty acid.

**Supplementary Table S4.**

Dose reduction index of cytarabine at fractions affected (Fa) = 0.9 in cytarabine-resistant cell lines.

|  | **THP-1** | **KG-1** |
| --- | --- | --- |
| Cytarabine + A939572 | 6.65 | 1.76 |
| Cytarabine + DS18561882 | 3.59 | 8.03 |

**Supplementary Table S5.**

Risk-correlation analysis of revised ELN2017 for genes in the Folate_metabolism pathway using multiomics databases.

| **Gene** |  | **OHSU** | | | |  | **TCGA-LAML** | **Proteomics** |
| --- | --- | --- | --- | --- | --- | --- | --- | --- |
|  | Jonckheere-Terpstra test | | Survival (DSS) | HR | Survival (OS) | HR | Jonckheere-Terpstra test | Jonckheere-Terpstra test |
| *ALDH1L1* | 0.965 | | 0.012 | 2.031 | 0.016 | 1.749 | 0.241 | 0.093 |
| *ALDH1L2* | 0.027 | | 0.004 | 2.303 | < 0.001 | 2.119 | < 0.001 | 0.001 |
| *DHFR* | 0.483 | | 0.001 | 3.075 | 0.002 | 2.029 | 0.800 | 0.259 |
| *DHFR2* | 0.995 | | 0.076 | 2.023 | 0.153 | 1.494 | 0.139 | NA |
| *FOLR2* | 0.353 | | 0.035 | 1.889 | 0.121 | 1.432 | < 0.001 | 0.002 |
| *FPGS* | 0.050 | | 0.002 | 0.414 | 0.003 | 0.525 | 0.406 | 0.424 |
| *MTHFD1* | 0.008 | | 0.035 | 1.865 | 0.009 | 2.149 | 0.004 | 0.275 |
| *MTHFD1L* | < 0.001 | | 0.043 | 1.805 | 0.056 | 1.561 | 0.003 | 0.816 |
| *MTHFD2* | < 0.001 | | 0.054 | 1.788 | 0.029 | 1.808 | 0.181 | 0.092 |
| *MTHFD2L* | 0.008 | | 0.051 | 0.553 | 0.236 | 0.759 | 0.747 | 0.500 |
| *MTHFR* | 0.017 | | 0.153 | 0.484 | 0.093 | 1.406 | < 0.001 | 0.061 |
| *MTHFS* | 0.269 | | 0.038 | 1.714 | 0.069 | 1.462 | 0.986 | 0.982 |
| *SHMT1* | 0.873 | | < 0.001 | 2.943 | 0.003 | 1.936 | 0.991 | 0.325 |
| *SHMT2* | 0.001 | | 0.007 | 2.103 | 0.004 | 2.014 | 0.158 | 0.339 |
| *SLC19A1* | 0.062 | | 0.066 | 1.639 | 0.060 | 1.490 | 0.001 | 0.002 |
| *SLC25A32* | 0.003 | | 0.049 | 5.777 | 0.073 | 2.741 | 0.034 | 0.014 |
| *SLC46A1* | 0.841 | | 0.072 | 1.780 | 0.235 | 1.363 | 0.111 | NA |

DSS, disease-specific survival; OS, overall survival; HR, the hazard ratio of the high expression group; NA, not available.

**References**

1. Tyner JW, Tognon CE, Bottomly D, et al. Functional genomic landscape of acute myeloid leukaemia. *Nature*. 2018;562(7728):526-531.

2. The Cancer Genome Atlas Research Network. Genomic and epigenomic landscapes of adult de novo acute myeloid leukemia. *N Engl J Med*. 2013;368(22):2059-2074.

3. Kramer MH, Zhang Q, Sprung R, et al. Proteomic and phosphoproteomic landscapes of acute myeloid leukemia. *Blood*. 2022;140(13):1533-1548.

4. Straube J, Ling VY, Hill GR, Lane SW. The impact of age, NPM1(mut), and FLT3(ITD) allelic ratio in patients with acute myeloid leukemia. *Blood*. 2018;131(10):1148-1153.

5. Hänzelmann S, Castelo R, Guinney J. GSVA: gene set variation analysis for microarray and RNA-seq data. *BMC Bioinformatics*. 2013;14:7.

6. Mizuno H, Kitada K, Nakai K, Sarai A. PrognoScan: a new database for meta-analysis of the prognostic value of genes. *BMC Med Genomics*. 2009;2:18.

7. Yoon S, Kim J, Kim SK, et al. GScluster: network-weighted gene-set clustering analysis. *BMC Genomics*. 2019;20(1):352.

8. Chou TC. Theoretical basis, experimental design, and computerized simulation of synergism and antagonism in drug combination studies. *Pharmacol Rev*. 2006;58(3):621-681.

9. Zhu F, Huang R, Li J, Liao X, Huang Y, Lai Y. Identification of Key Genes and Pathways Associated with RUNX1 Mutations in Acute Myeloid Leukemia Using Bioinformatics Analysis. *Med Sci Monit*. 2018;24:7100-7108.

10. Huang R, Liao X, Li Q. Identification of key pathways and genes in TP53 mutation acute myeloid leukemia: evidence from bioinformatics analysis. *Onco Targets Ther*. 2018;11:163-173.

11. Guo C, Gao YY, Ju QQ, Zhang CX, Gong M, Li ZL. The landscape of gene co-expression modules correlating with prognostic genetic abnormalities in AML. *J Transl Med*. 2021;19(1):228.

12. Lai B, Lai Y, Zhang Y, Zhou M, OuYang G. Survival prediction in acute myeloid leukemia using gene expression profiling. *BMC Med Inform Decis Mak*. 2022;22(1):57.

13. Tang Y, Xiao S, Wang Z, et al. A Prognostic Model for Acute Myeloid Leukemia Based on IL-2/STAT5 Pathway-Related Genes. *Front Oncol*. 2022;12:785899.

14. Pabst T, Kortz L, Fiedler GM, Ceglarek U, Idle JR, Beyoğlu D. The plasma lipidome in acute myeloid leukemia at diagnosis in relation to clinical disease features. *BBA Clin*. 2017;7:105-114.

15. Bonagas N, Gustafsson NMS, Henriksson M, et al. Pharmacological targeting of MTHFD2 suppresses acute myeloid leukemia by inducing thymidine depletion and replication stress. *Nat Cancer*. 2022;3(2):156-172.

16. Döhner H, Wei AH, Appelbaum FR, et al. Diagnosis and management of AML in adults: 2022 recommendations from an international expert panel on behalf of the ELN. *Blood*. 2022;140(12):1345-1377.

17. Jentzsch M, Bischof L, Ussmann J, et al. Prognostic impact of the AML ELN2022 risk classification in patients undergoing allogeneic stem cell transplantation. *Blood Cancer J*. 2022;12(12):170.
